# Long-Term Outcomes According to the Initially Intended Stenting Strategy for Unprotected Distal Left Main Bifurcation PCI: A Single-Centre Retrospective Cohort Study

**DOI:** 10.3390/jcm15207817

**Published:** 2026-10-10

**Authors:** Zafer Guven, Hidayet Ozan Arabaci, Hakan Yalman, Melike Zeynep Dursun, Ibrahim Oguz Karaca

**Affiliations:** 1 Gaziosmanpasa Training and Research Hospital, 34255 Istanbul, Turkey; drzaferguven80@gmail.com (Z.G.); hakan.yalman.96@gmail.com (H.Y.); 2 Yuksekova State Hospital, 30300 Hakkari, Turkey; 3 Medipol Mega University Hospital, 34214 Istanbul, Turkeyoguzkaraca@hotmail.com (I.O.K.)

**Keywords:** left main coronary artery disease, coronary bifurcation, percutaneous coronary intervention, provisional stenting, DK-crush, intravascular ultrasound, intra-aortic balloon pump

## Abstract

**Background:** Optimal stenting for distal unprotected left main coronary artery (LMCA) bifurcation disease remains uncertain. We evaluated long-term outcomes by initially intended strategy. **Methods:** This retrospective, single-centre cohort included 234 consecutive patients undergoing percutaneous coronary intervention (PCI) without acute coronary syndrome, classified by intended strategy irrespective of crossover. Cox models for all-cause mortality were adjusted for strategy, age, left ventricular ejection fraction, SYNTAX score, and diabetes, with anatomical and propensity-score sensitivity analyses. Secondary and subgroup analyses were exploratory. **Results:** Provisional and upfront two-stent strategies were intended in 145 and 89 patients. Two-stent patients had greater anatomical complexity. During median 49-month follow-up, mortality did not differ significantly (two-stent versus provisional: 16.9% versus 21.4%; *p* = 0.499; adjusted hazard ratio, 0.83; 95% confidence interval, 0.43–1.60; *p* = 0.579), with consistent sensitivity analyses. Older age, lower ejection fraction, and higher SYNTAX score were associated with mortality. Target-lesion revascularisation (7.8% versus 2.1%; *p* = 0.033) and in-hospital stroke (5.6% versus 0.7%; *p* = 0.031) were more frequent with intended two-stent PCI; other secondary outcomes did not differ significantly. Exploratory unadjusted comparisons showed lower target-lesion and target-vessel revascularisation with double-kissing crush (n = 74) versus conventional culotte (n = 15), limited by small samples and selection bias. Intravascular ultrasound was associated with lower mortality in exploratory adjusted analyses. Mortality was numerically lower with elective intra-aortic balloon pump support at ejection fraction ≤40%, without statistical significance. **Conclusions:** No, statistically significant association between initially intended bifurcation strategy and long-term all-cause mortality was observed. Limited precision precludes an inference of equivalence, and exploratory differences in target-lesion revascularisation and in-hospital stroke warrant caution. These findings provide a long-term real-world perspective on individualised distal LMCA PCI, integrating lesion complexity, ventricular reserve, procedural technique, and intravascular imaging.

## 1. Introduction

Significant left main coronary artery (LMCA) disease has major prognostic implications because it jeopardises a large myocardial territory and therefore warrants revascularisation when clinically significant [1,2]. Although coronary artery bypass grafting (CABG) remains the reference strategy for many patients, percutaneous coronary intervention (PCI) with contemporary drug-eluting stents is an accepted alternative after multidisciplinary Heart Team assessment in appropriately selected patients, particularly when coronary anatomy is suitable and anatomical complexity is low to intermediate [1,2,3]. An individual patient-data meta-analysis of four randomised trials found no significant difference in 5-year all-cause mortality between PCI and CABG, while confirming distinct profiles of spontaneous myocardial infarction, procedural myocardial infarction, stroke, and repeat revascularisation that favour individualised decision-making [3].

Once PCI is selected, distal LMCA bifurcation anatomy adds substantial technical and prognostic complexity [4]. A stepwise provisional strategy minimises stent length, metal burden, and procedural complexity and remains the default approach for most bifurcation lesions [4,5]. However, complex true bifurcations with extensive disease of a clinically important left circumflex branch may require an upfront two-stent strategy, with double-kissing crush (DK-crush) supported by dedicated randomised evidence in appropriately selected anatomy [4,6,7]. At 3 years, DKCRUSH-V showed lower target lesion failure and stent thrombosis with DK-crush than with provisional stenting in true distal LMCA bifurcations, while DEFINITION II favoured systematic two-stent treatment in bifurcations meeting prespecified criteria for anatomical complexity [6,7]. Conversely, EBC MAIN found no significant reduction in major adverse cardiac events with systematic dual stenting at 1 year and subsequently reported less clinically driven target lesion revascularisation with the stepwise provisional strategy at 3 years [5,8]. These apparently divergent findings suggest that outcomes depend not on the universal superiority of one technique, but on lesion complexity, side-branch disease burden, the selected two-stent method, and operator expertise [4,5,6,7,8].

Beyond bifurcation strategy, procedural optimisation with intravascular ultrasound (IVUS) and haemodynamic support in selected patients may influence outcomes after complex LMCA PCI [1,9,10,11]. IVUS enables detailed lesion characterisation, appropriate stent sizing, and assessment of stent expansion and apposition and is recommended to guide complex PCI, particularly in LMCA disease [1,9]. Elective intra-aortic balloon pump (IABP) support may provide temporary haemodynamic stability during high-risk PCI, but BCIS-1 showed no reduction in early major adverse cardiac and cardiovascular events, although its long-term follow-up showed lower all-cause mortality; therefore, its net clinical benefit remains uncertain [10,11].

Against this background, the present study evaluated long-term clinical outcomes according to the initially intended bifurcation stenting strategy in a consecutive, single-centre, retrospective cohort of adults undergoing PCI for unprotected LMCA disease outside the setting of an acute coronary syndrome. The primary objective was to compare long-term all-cause mortality between patients categorised according to an initially intended provisional or upfront two-stent strategy, irrespective of subsequent crossover. Secondary objectives were to compare other clinical outcomes and to explore findings according to true bifurcation status, the two-stent technique used, left ventricular systolic function, IVUS use, and elective IABP support.

## 2. Materials and Methods

### 2.1. Study Population and Design

This single-centre, retrospective observational cohort study included 234 consecutive adults who underwent percutaneous coronary intervention (PCI) for unprotected distal left main coronary artery (LMCA) bifurcation disease at the Department of Cardiology, Medipol University Faculty of Medicine Hospital, between 11 December 2019 and 20 November 2021.

Patients were eligible if they were aged ≥18 years and had clinically significant LMCA disease requiring revascularisation. Significant LMCA disease was defined as a visually estimated diameter stenosis of ≥50% on coronary angiography. In patients with angiographically intermediate LMCA lesions (20–49% diameter stenosis), intravascular ultrasound (IVUS) was performed, and a minimal lumen area of <6.0 mm^2^ was considered indicative of significant stenosis [12,13]. The indication for revascularisation was determined according to symptoms, objective evidence of myocardial ischaemia, angiographic findings, and, when applicable, IVUS measurements.

Patients presenting with an acute coronary syndrome or with a history of CABG or previous LMCA PCI were not eligible. Unprotected LMCA disease was defined as LMCA disease without a patent bypass graft to the left anterior descending or left circumflex coronary artery. Patients with any previous CABG were excluded from this cohort.

During the study period, 293 patients with unprotected LMCA disease were evaluated for revascularisation. Of these, 59 were excluded: 37 were directed to CABG following Heart Team assessment, 7 had isolated ostial or shaft LMCA lesions without distal bifurcation involvement, 8 had no available follow-up data, and 7 had insufficient clinical or procedural data for analysis. The final analytical cohort comprised 234 patients who underwent PCI for unprotected distal LMCA bifurcation disease. Patient selection is illustrated in Figure 1.

All coronary angiograms were reviewed by at least two experienced interventional cardiologists, and anatomical complexity was assessed using the validated SYNTAX score algorithm [14]. Following diagnostic angiography, the revascularisation strategy was evaluated by a multidisciplinary heart team comprising interventional cardiologists and cardiovascular surgeons, in accordance with the 2018 European Society of Cardiology/European Association for Cardio-Thoracic Surgery myocardial revascularisation guidelines [13]. Both PCI and coronary artery bypass grafting were considered in patients with low-to-intermediate anatomical complexity. Surgical revascularisation was preferred in patients with high anatomical complexity when clinically feasible, whereas PCI was performed in patients considered to be at high surgical risk or in those who declined surgery after being informed of the available treatment options.

Baseline demographic characteristics, cardiovascular comorbidities, laboratory measurements, angiographic findings, procedural characteristics, and follow-up data were obtained from the institutional electronic medical records and the national healthcare database.

The study protocol was approved by the Clinical Research Ethics Committee of Medipol University Faculty of Medicine (approval no. 10848898-604.01.01-E.19389; 3 January 2022) and was conducted in accordance with the Declaration of Helsinki. All patients provided written informed consent for the index PCI. Under institutional policy, the requirement for additional informed consent for retrospective study participation was waived following ethics committee approval.

### 2.2. Percutaneous Coronary Intervention

All procedures were performed in accordance with contemporary interventional practice. Before PCI, patients who had not received maintenance P2Y12 inhibitor therapy for at least 5 days were administered a loading dose of a P2Y12 inhibitor. Clopidogrel was the preferred agent and was administered at a loading dose of 600 mg, followed by a maintenance dose of 75 mg once daily. Ticagrelor, at a loading dose of 180 mg followed by 90 mg twice daily, or prasugrel, at a loading dose of 60 mg followed by 10 mg once daily, was used at the operator’s discretion. Patients also received aspirin at a loading dose of 300 mg or continued maintenance therapy with 100 mg once daily. Dual antiplatelet therapy was generally planned for 12 months after PCI, with duration individualised according to ischaemic and bleeding risks [15]. Unfractionated heparin was administered during the procedure to maintain an activated clotting time of >250 s, and glycoprotein IIb/IIIa inhibitors were reserved for selected cases at the operator’s discretion.

Radial access was routinely preferred, and the LMCA was generally engaged using a 7-Fr extra-backup guiding catheter. All lesions underwent predilatation and lesion preparation. Compliant balloons were used in most procedures, whereas non-compliant, cutting, or scoring balloons were selected according to lesion morphology and complexity. Post-dilatation was performed using non-compliant balloons [4,16].

When intravascular imaging was used, IVUS was performed with an OptiCross catheter (Boston Scientific, Marlborough, MA, USA). IVUS was routinely used to determine the indication for revascularisation in angiographically intermediate LMCA lesions with 20–49% diameter stenosis. During PCI, IVUS was used to guide stent sizing and evaluate stent expansion and apposition. Routine post-PCI IVUS was not mandated, and its use was determined by the operator according to lesion characteristics and procedural requirements. For the study analyses, procedures in which IVUS was used for lesion assessment and/or stent optimisation were classified as IVUS-guided PCI.

The stenting strategy was selected by at least two experienced interventional cardiologists according to the Medina classification, side-branch lesion length, and overall lesion complexity. A provisional strategy was generally selected for non-true bifurcation lesions and for true bifurcations with a side-branch lesion length of <5 mm [4,16]. Only lesions involving the distal LMCA bifurcation were included; isolated ostial or shaft LMCA lesions without distal bifurcation involvement were excluded. Distal LMCA bifurcation lesions were classified using the Medina classification. True bifurcation status was determined retrospectively from angiographic review and was defined as Medina 1,1,1, 1,0,1, or 0,1,1 [17]. An upfront two-stent strategy was used when lesion anatomy was considered unsuitable for a provisional approach. Patients were classified according to the initially intended stenting strategy. Accordingly, patients who required an unplanned side-branch stent after an initially provisional approach remained in the provisional-strategy group. When crossover to a second stent was required, T and protrusion, culotte, or reverse-crush techniques were used. Among patients assigned to an upfront two-stent strategy, DK-crush was the most frequently used technique, followed by culotte. Patient-level data on proximal optimisation technique (POT), final kissing-balloon inflation, re-POT, separate side-branch predilatation/post-dilatation, and post-PCI IVUS measurements, including minimal stent area, were unavailable for analysis. Drug-eluting stents were implanted in all patients, including everolimus-, sirolimus-, zotarolimus-, and biolimus-eluting stents. Complete revascularisation was pursued whenever clinically feasible, with staged procedures performed when appropriate.

Intra-aortic balloon pump (IABP) support was not used routinely and was initiated at the operator’s discretion. Elective IABP support was considered before PCI in patients considered to be at high risk of haemodynamic compromise, including those with severe left ventricular systolic dysfunction, a large myocardial territory at risk, or anticipated prolonged ischaemia during complex distal LMCA bifurcation PCI. Bailout IABP support was used in the event of clinically significant intraprocedural haemodynamic deterioration, including persistent hypotension, slow-flow or no-reflow with ongoing ischaemia, acute pulmonary oedema, sustained ventricular arrhythmia with low cardiac output, circulatory instability, or evolving cardiogenic shock [10,18].

### 2.3. Laboratory Findings and Echocardiography

Baseline laboratory measurements obtained during the index hospitalisation were retrieved from the institutional electronic medical records. When necessary, the available clinical and laboratory information was supplemented using records from the national healthcare database.

Transthoracic echocardiography was performed during the index hospitalisation by two experienced operators using an ultrasound system, in accordance with the 2015 recommendations of the American Society of Echocardiography and the European Association of Cardiovascular Imaging for cardiac chamber quantification. Standard parasternal and apical views were acquired. Left ventricular ejection fraction was calculated from left ventricular end-diastolic and end-systolic volumes measured in the apical four- and two-chamber views using the biplane method of disks (modified Simpson method) [19].

### 2.4. Clinical Follow-Up, Endpoints and Definitions

Patients who survived to hospital discharge underwent clinical follow-up at 1 week and at 1, 3, and 6 months after the index procedure, and annually thereafter. Follow-up information was obtained through outpatient clinic visits or structured telephone interviews. For patients who could not be contacted, vital status and subsequent hospital admissions were ascertained from national electronic healthcare records and public health registries. Patients were followed until death or the most recent documented clinical contact.

The primary endpoint was all-cause mortality during follow-up. Secondary endpoints included cardiac death, non-fatal myocardial infarction (MI), in-hospital mortality, periprocedural MI, target lesion revascularisation (TLR), target vessel revascularisation (TVR), in-hospital stroke, contrast-associated acute kidney injury (CA-AKI), and procedural success. Major adverse cardiovascular events (MACE) were defined as a study-specific composite of cardiac death, post-discharge non-fatal MI, and in-hospital stroke. This study-specific composite combined recorded cardiac death, post-discharge non-fatal MI, and in-hospital stroke. Periprocedural MI was evaluated separately to distinguish procedural MI from MI occurring after discharge. This definition should be considered when comparing MACE with composites used in other studies. Patients who experienced more than one component of the MACE composite were counted only once in the composite endpoint.

Deaths were classified as cardiac unless an unequivocal non-cardiac cause was established. MI, including periprocedural MI, was defined according to the Fourth Universal Definition of Myocardial Infarction [20]. TLR was defined as repeat percutaneous revascularisation for restenosis or another complication involving the target lesion. The target lesion comprised the treated segment, including the stented segment and the 5 mm margins proximal and distal to the stent. TVR was defined as any repeat percutaneous revascularisation involving any segment of the target vessel, including the target lesion [21]. Stroke analyses were restricted to events recorded during the index hospitalisation. Post-discharge stroke data were unavailable for analysis.

CA-AKI was defined according to the Kidney Disease: Improving Global Outcomes criteria as an increase in serum creatinine of ≥0.3 mg/dL within 48 h or to ≥1.5 times the baseline value within 7 days after contrast exposure [22]. Procedural success was defined as residual stenosis <30% with TIMI grade 3 flow and no in-hospital death, periprocedural MI, stroke, or emergency repeat revascularisation.

All clinical events were independently adjudicated through medical-record review by cardiologists who were not involved in the index procedure.

### 2.5. Statistical Analysis

Statistical analyses were performed using Python 3 (version 3.11) with the SciPy (version 1.18) and statsmodels libraries.

The additional sensitivity analyses were implemented in Python 3 (version 3.12) using NumPy, SciPy, and scikit-learn. The distributions of continuous variables were assessed using the Shapiro–Wilk test and visual inspection. Continuous variables are presented as mean ± standard deviation when approximately normally distributed and as median with interquartile range otherwise. Categorical variables are presented as number and percentage. Between-group comparisons were performed using Student’s independent-samples *t*-test or the Mann–Whitney U test for continuous variables and Pearson’s chi-square test or Fisher’s exact test for categorical variables, as appropriate. Between-group differences in principal baseline and procedural characteristics were additionally quantified using standardised mean differences (SMDs). Their absolute values were used to describe the magnitude of imbalance independently of the between-group *p* values.

Survival time was calculated from the date of the index PCI to death or the most recent documented clinical contact. Cumulative survival probabilities were estimated using the Kaplan–Meier method and compared between groups using the log-rank test. Secondary non-fatal outcomes were summarised as the proportions of patients with recorded events during their available follow-up. Event dates were unavailable in the analytical dataset, precluding time-to-first-event or fixed-time cumulative-incidence estimation. These crude proportions do not account for differences in follow-up duration or competing death.

A multivariable Cox proportional-hazards model was constructed to evaluate the association between initially intended stenting strategy and all-cause mortality, adjusting for age, LVEF, SYNTAX score, and diabetes mellitus. These covariates were selected a priori to represent clinical risk and overall anatomical complexity. The primary model was limited to five covariates given the 46 observed deaths. The provisional strategy was the reference category, and age, LVEF, and SYNTAX score were entered as continuous variables. Hazard ratios are reported with 95% confidence intervals.

Exploratory sensitivity analyses were performed to address measured treatment-selection factors. First, the primary Cox model was additionally adjusted for true bifurcation status. Second, a propensity score for intended upfront two-stent PCI was estimated using logistic regression including age, sex, diabetes mellitus, hypertension, LVEF, eGFR, SYNTAX score, and true bifurcation status. Intended strategy and the propensity score, entered as a continuous covariate, were then included in a Cox model. This analysis was repeated within common support, defined as the intersection of the observed propensity-score ranges in the two strategy groups, retaining the scores estimated in the full cohort. Tied event times were handled using the Breslow method. Proportional hazards were assessed using Schoenfeld residual-based score tests with a Kaplan–Meier time transformation. Following a signal of non-proportionality for age, an additional model including true bifurcation status allowed the age effect to vary with transformed time. An exploratory Cox model assessed the association between IVUS use and all-cause mortality, adjusting for initially intended strategy, age, LVEF, SYNTAX score, diabetes mellitus, and true bifurcation status. A time-varying age effect was included using the same Kaplan–Meier-transformed-time interaction described above.

Exploratory subgroup analyses were performed according to true bifurcation status, the two-stent technique used, left ventricular ejection fraction ≤40%, IVUS use, and elective IABP support. Among patients with an LVEF ≤40%, outcomes were compared between those receiving elective IABP support and those undergoing PCI without IABP support; patients requiring bailout IABP were excluded from this comparison. Because these analyses were exploratory and involved relatively small subgroups, no adjustment was made for multiple comparisons; their findings should therefore be interpreted as hypothesis-generating.

No imputation was performed. Intended strategy, mortality status, follow-up duration, and all covariates used in the primary Cox, propensity-score, and exploratory IVUS mortality models were available for all 234 included patients. True bifurcation status, two-stent technique among the 89 upfront two-stent patients, IVUS use, LVEF category, and elective/bailout IABP status were also available for all relevant patients; no additional exclusions were required because of missing subgroup-classification data. All statistical tests were two-sided, and a *p* value < 0.05 was considered statistically significant.

## 3. Results

A total of 234 patients who underwent PCI for unprotected LMCA disease were included in the study. All included patients underwent PCI for unprotected distal LMCA bifurcation disease; no patients with isolated ostial or shaft LMCA lesions were included. True bifurcation lesions, defined as Medina 1,1,1, 1,0,1, or 0,1,1, were present in 104 patients (44.4%), while the remaining 130 patients (55.6%) had non-true bifurcation lesions. The initially intended strategy was provisional stenting in 145 patients (62.0%) and upfront two-stent treatment in 89 patients (38.0%). Among patients assigned to an upfront two-stent strategy, DK-crush was used in 74 (83.1%) and conventional culotte in 15 (16.9%). An unplanned side-branch stent was required in 21 patients (14.5%) initially assigned to the provisional strategy; these patients remained in the provisional group for all analyses. Radial access was used in 189 patients (80.8%), and IVUS was used in 128 procedures (54.7%). The median follow-up duration was 49 months (interquartile range, 43–53 months).

Baseline and procedural characteristics according to the initially intended stenting strategy are presented in Table 1. The groups did not differ significantly in age, sex, cardiovascular comorbidities, left ventricular ejection fraction, estimated glomerular filtration rate, or haemoglobin concentration. Patients assigned to the upfront two-stent strategy had a higher mean SYNTAX score than those assigned to the provisional strategy (32.1 ± 4.1 vs. 27.0 ± 6.4; *p* < 0.001) and more frequently had true bifurcation lesions (84.3% vs. 20.0%; *p* < 0.001). Overall, MACE occurred in 39 patients (16.7%). The component counts comprised 19 cardiac deaths (8.1%), 15 non-fatal MIs occurring after hospital discharge (6.4%), and six in-hospital strokes (2.6%). One patient in the upfront two-stent group had both in-hospital stroke and cardiac death and was counted once in the composite, accounting for the difference between the summed component count of 40 and the 39 patients with MACE. Periprocedural MI occurred in nine patients (3.8%) and was reported separately, without inclusion in the MACE composite.

SMDs indicated marked anatomical differences between the intended-strategy groups, particularly in SYNTAX score and true bifurcation status, with absolute SMDs of 0.95 and 1.68, respectively. Substantial differences were also observed in IVUS use (absolute SMD 1.51). SMDs for the principal baseline and procedural characteristics are presented in Supplementary Table S1.

IVUS was used more frequently in the two-stent group than in the provisional group (91.0% vs. 32.4%; *p* < 0.001). The two-stent group also had a greater number of implanted stents (3.1 ± 0.9 vs. 2.1 ± 0.9; *p* < 0.001), a longer total stent length (92.1 ± 28.5 mm vs. 55.3 ± 26.4 mm; *p* < 0.001), and more frequent side-branch stent implantation (100.0% vs. 14.5%; *p* < 0.001). Procedural success did not differ statistically between the two groups (92.1% vs. 91.0%; *p* = 0.959). No-reflow occurred in five patients (3.4%) in the provisional group and in none of the patients in the two-stent group (*p* = 0.160).

During a median follow-up of 49 months, 46 patients (19.7%) died. All-cause mortality was observed in 15 patients (16.9%) assigned to the upfront two-stent strategy and 31 patients (21.4%) assigned to the provisional strategy (*p* = 0.499). Consistent with this finding, Kaplan–Meier analysis showed no significant difference in overall survival between the two strategies (log-rank *p* = 0.415; Figure 2).

Cardiac mortality did not differ statistically between the two-stent and provisional groups (6.7% vs. 9.0%; *p* = 0.720). MACE occurred in 16 patients (18.0%) in the two-stent group and 23 patients (15.9%) in the provisional group (*p* = 0.810). In-hospital mortality and non-fatal MI during follow-up also did not differ significantly between the groups.

All secondary-outcome and subgroup comparisons were exploratory, with nominal *p* values unadjusted for multiple comparisons. In-hospital stroke was more frequent in the two-stent group than in the provisional group (5.6% vs. 0.7%; *p* = 0.031). Periprocedural MI was numerically more frequent in the two-stent group, although the between-group difference did not reach statistical significance (6.7% vs. 2.1%; *p* = 0.087). TLR was observed in 7.8% of patients in the two-stent group and 2.1% in the provisional group (*p* = 0.033). The rates of TVR (10.1% vs. 6.2%; *p* = 0.316) and CA-AKI (4.5% vs. 6.2%; *p* = 0.771) did not differ significantly between the groups. Clinical outcomes according to the initially intended bifurcation strategy are summarised in Table 2.

In the multivariable Cox proportional-hazards model, older age, lower left ventricular ejection fraction, and a higher SYNTAX score were independently associated with all-cause mortality (Table 3). Each 1-year increase in age was associated with a higher mortality hazard (adjusted hazard ratio [HR], 1.07; 95% confidence interval [CI], 1.04–1.10; *p* < 0.001). Conversely, each 1-percentage-point increase in left ventricular ejection fraction was associated with a lower mortality hazard (adjusted HR, 0.95; 95% CI, 0.92–0.97; *p* < 0.001). A higher SYNTAX score was also associated with increased mortality (adjusted HR per 1-point increase, 1.11; 95% CI, 1.05–1.18; *p* < 0.001).

No statistically significant association between initially intended two-stent treatment and all-cause mortality was observed (adjusted HR, 0.83; 95% CI, 0.43–1.60; *p* = 0.579). Similarly, diabetes mellitus was not independently associated with mortality in the adjusted model (adjusted HR, 1.12; 95% CI, 0.61–2.07; *p* = 0.719). The adjusted associations are presented graphically in Figure 3.

Sensitivity analyses yielded similar findings (Table S2). Additional adjustment for true bifurcation status gave an HR of 0.84 (95% CI, 0.39–1.83; *p* = 0.665). The propensity model had a c-statistic of 0.878, and common support included 215 patients with 41 deaths. The propensity-score-adjusted HR was 0.71 (95% CI, 0.31–1.62; *p* = 0.412) in the full cohort and 0.73 (95% CI, 0.32–1.67; *p* = 0.451) within common support. Global proportional-hazards tests were not statistically significant, although individual diagnostics indicated non-proportionality for age in the primary and anatomy-adjusted models (both *p* = 0.040). Allowing a time-varying age effect yielded an HR of 0.85 (95% CI, 0.39–1.84; *p* = 0.679) for intended two-stent treatment.

Among the 104 patients with true bifurcation lesions, 29 were initially assigned to the provisional strategy and 75 to an upfront two-stent strategy. No statistically significant differences in clinical outcomes were observed between the two strategies in this subgroup (Table 4).

All-cause mortality was numerically lower in the two-stent group than in the provisional group (17.3% vs. 34.5%; *p* = 0.104), as were cardiac mortality (6.7% vs. 20.7%; *p* = 0.069) and MACE (17.3% vs. 31.0%; *p* = 0.205). Conversely, TLR (9.3% vs. 0%; *p* = 0.186) and TVR (12.0% vs. 0%; *p* = 0.059) were numerically more frequent in the two-stent group. None of these differences reached statistical significance.

Among the 89 patients initially assigned to an upfront two-stent strategy, DK-crush was used in 74 and conventional culotte stenting in 15. No statistically significant differences were observed between the two technique groups in age, left ventricular ejection fraction, SYNTAX score, prevalence of true bifurcation lesions, or total stent length (Table 5).

All-cause mortality was observed in 14 patients (18.9%) treated with DK-crush and one patient (6.7%) treated with conventional culotte stenting (*p* = 0.248). Cardiac mortality did not differ statistically between the groups (6.8% vs. 6.7%; *p* = 1.000). Both TLR (4.1% vs. 26.7%; *p* = 0.014) and TVR (6.7% vs. 26.7%; *p* = 0.040) were more frequent with conventional culotte stenting than with DK-crush. MACE was also numerically more frequent with culotte stenting, although the difference was not statistically significant (20.0% vs. 17.6%; *p* = 0.729).

Patients were stratified according to a left ventricular ejection fraction of ≤40% or >40%. Of the 234 patients, 40 had a left ventricular ejection fraction of ≤40% and 194 had a left ventricular ejection fraction of >40% (Table 6).

All-cause mortality was higher among patients with a left ventricular ejection fraction of ≤40% than among those with a left ventricular ejection fraction of >40% (55.0% vs. 12.4%; *p* < 0.001). Similarly, cardiac mortality (32.5% vs. 3.1%; *p* < 0.001) and MACE (37.5% vs. 12.4%; *p* < 0.001) were more frequent in patients with a left ventricular ejection fraction of ≤40%. These findings were consistent with the inverse association between left ventricular ejection fraction and all-cause mortality observed in the multivariable Cox model.

IVUS was used in 128 procedures (54.7%), whereas 106 procedures were performed without IVUS. The unadjusted clinical outcomes according to IVUS use are presented in Table 7.

All-cause mortality was lower among patients who underwent IVUS-guided PCI than among those who underwent PCI without IVUS (10.9% vs. 30.2%; *p* < 0.001). Kaplan–Meier analysis also demonstrated a difference in overall survival between the groups (log-rank *p* < 0.001; Figure 4). MACE was less frequent in the IVUS-guided group (10.2% vs. 24.5%; *p* = 0.006).

Cardiac mortality was numerically lower with IVUS-guided PCI, although the difference did not reach statistical significance (4.7% vs. 12.3%; *p* = 0.061). TLR rates did not differ statistically between patients undergoing PCI with and without IVUS guidance (4.7% vs. 3.8%; *p* = 0.731). IVUS-treated patients had higher LVEF and SYNTAX scores, more frequent true bifurcation disease and intended two-stent treatment, and greater stent number and length (Table S3). In the exploratory adjusted model, IVUS use remained associated with lower mortality (HR, 0.28; 95% CI, 0.14–0.56; *p* < 0.001).

Intra-aortic balloon pump (IABP) support was used in 15 patients: electively in 8 and as bailout support in 7. Among the 40 patients with an LVEF ≤40%, 8 received elective IABP support, 3 required bailout IABP because of intraprocedural haemodynamic deterioration, and 29 underwent PCI without IABP support. To distinguish the outcomes associated with planned haemodynamic support from those associated with rescue therapy, patients receiving bailout IABP were excluded from the comparison between elective IABP support and no IABP support.

Age, LVEF, and SYNTAX score did not differ significantly between patients receiving elective IABP support and those undergoing PCI without IABP support.

All-cause mortality was numerically lower among patients receiving elective IABP support than among those undergoing PCI without IABP support (2/8 [25.0%] vs. 18/29 [62.1%]; *p* = 0.109). Cardiac mortality was also numerically lower in the elective-IABP group (0/8 [0.0%] vs. 11/29 [37.9%]; *p* = 0.076). In-hospital mortality did not differ between the groups (0/8 [0.0%] vs. 2/29 [6.9%]; *p* = 1.000). MACE was numerically less frequent in the elective-IABP group, although the difference was not statistically significant (1/8 [12.5%] vs. 11/29 [37.9%]; *p* = 0.342). Conversely, contrast-associated acute kidney injury occurred numerically more frequently among patients receiving elective IABP support, although the difference was not statistically significant (2/8 [25.0%] vs. 4/29 [13.8%]; *p* = 0.591). These exploratory findings should be interpreted cautiously because of the small subgroup size and the non-randomised selection of patients for elective IABP support (Figure 5).

## 4. Discussion

In this real-world cohort of 234 patients undergoing PCI for unprotected distal LMCA bifurcation disease outside the setting of acute coronary syndrome, we evaluated outcomes according to the initially intended bifurcation strategy over a median follow-up of 49 months. Patients selected for upfront two-stent PCI had substantially greater anatomical complexity, including higher SYNTAX scores and a markedly higher prevalence of true bifurcation lesions. No statistically significant association between intended strategy and long-term all-cause mortality was observed in the primary adjusted model (HR, 0.83; 95% CI, 0.43–1.60), with consistent findings across anatomical and propensity-score sensitivity analyses. Nevertheless, the 46 observed deaths and wide confidence intervals indicate limited precision; these findings do not establish equivalence or exclude clinically meaningful benefit or harm. The higher observed proportions of TLR and in-hospital stroke among patients selected for two-stent PCI warrant cautious interpretation because these exploratory comparisons remain susceptible to confounding by indication and multiple testing. Analyses of true bifurcation status, stenting technique, IVUS use, and elective IABP support, including the exploratory adjusted IVUS model, offer complementary clinical context but cannot establish comparative treatment efficacy. By retaining the initially intended strategy despite subsequent crossover, this study provides a clinically relevant framework for interpreting long-term outcomes and procedural decision-making in patients with heterogeneous distal LMCA anatomy.

The absence of a statistically significant survival difference between the intended strategies is consistent with contemporary evidence. EBC MAIN showed no significant difference in death or MI between stepwise provisional and systematic dual-stent treatment at either 1 or 3 years, although clinically driven TLR was lower with the provisional approach at extended follow-up [5,8]. In the distal LMCA cohort of EXCEL, the advantage associated with provisional PCI was largely confined to lesions without substantial involvement of both distal branches [23]. Meta-analyses have likewise shown no consistent difference in mortality or MI between provisional and systematic two-stent strategies, whereas differences in composite outcomes have been driven predominantly by TLR or TVR [24,25]. These observations suggest that stent number alone has limited influence on survival, whereas lesion-related failure is more sensitive to anatomical complexity and the durability of bifurcation reconstruction.

Anatomical complexity provides an important framework for reconciling apparently conflicting trials. DKCRUSH-V demonstrated lower target lesion failure with planned DK-crush than with provisional treatment in true distal LMCA bifurcations at both 1 and 3 years, principally through reductions in target-vessel MI, TLR, and stent thrombosis rather than cardiac mortality [6,26]. Compared with EBC MAIN, DKCRUSH-V enrolled more complex lesions with longer side-branch disease and a higher crossover rate, and its planned two-stent technique was specifically DK-crush rather than predominantly culotte or T/TAP [5,8,26]. Likewise, DEFINITION II showed that planned two-stent PCI reduced target lesion failure in prespecified complex bifurcations, with the benefit persisting at 3 years [7,27,28]. Our true bifurcation analysis was directionally compatible with this complexity-based model: mortality and MACE were numerically lower with upfront two-stent PCI, whereas TLR and TVR were numerically higher. However, the small provisional subgroup (n = 29), unadjusted comparisons, and multiple testing preclude conclusions about equivalence, benefit, or effect modification.

The higher overall TLR rate after intended two-stent PCI should also be interpreted in the context of confounding by indication. Patients selected for dual stenting had higher SYNTAX scores, a more than fourfold greater prevalence of true bifurcation disease and consequently received more stents with greater total stent length. Furthermore, the EBC MAIN restenosis analysis identified the ostial LCx as the predominant site of recurrent disease leading to TLR, underlining the intrinsic vulnerability of this anatomical segment [29]. Thus, the observed TLR difference may partly reflect the disease burden prompting selection of two-stent PCI rather than a detrimental effect of the strategy itself. Classification according to the initially intended strategy preserved the original procedural decision and avoided outcome-dependent reclassification of crossover cases, but it could not eliminate the anatomical imbalance inherent in operator-selected treatment. The SMD analysis quantified the substantial anatomical differences between the intended-strategy groups, particularly in SYNTAX score and true bifurcation status, together with differences in concomitant IVUS use. These findings highlight treatment-selection differences and limit attribution of the observed clinical outcomes to stenting strategy alone. The anatomy- and propensity-adjusted mortality analyses addressed selected measured treatment-selection factors but did not eliminate residual confounding, including the marked difference in IVUS use. Their wide confidence intervals remained compatible with clinically meaningful benefit or harm and therefore do not establish equivalence.

The specific two-stent technique may be as important as the decision to implant a second stent. In our cohort, DK-crush was associated with lower TLR and TVR than conventional culotte, consistent with DKCRUSH-III and network meta-analyses in which DK-crush performed favourably for lesion- and vessel-related outcomes without a consistent mortality advantage [30,31,32,33]. Conventional culotte, however, should not be equated with contemporary DK-culotte. By adding a kissing-balloon inflation after side-branch stenting and before main-vessel stent deployment, followed by a second final kissing inflation, DK-culotte achieved overall malapposition comparable to conventional culotte but lower than DK-crush in bench testing [34]. This distinction is supported by emerging clinical evidence. In the prospective, non-randomised ROUTE study of 202 non-left-main bifurcations, DK-culotte was associated with lower 1-year target lesion failure than DK-crush [35]. In the nine-centre MAIN-ROUTE registry of 245 true distal LMCA bifurcations, 3-year target lesion failure was numerically lower with DK-culotte than with DK-crush (5.0% vs. 11.3%; *p* = 0.06), without significant differences in TLR, target-vessel MI, or cardiac mortality [36]. These observational data do not establish DK-culotte superiority but indicate that the advantage of DK-crush over conventional culotte should not be extrapolated to the contemporary DK modification. Accordingly, our technique-specific findings remain hypothesis-generating, particularly because only 15 patients underwent culotte stenting, and reinforce the importance of anatomy, technical optimisation, and operator expertise emphasised by current consensus recommendations [37].

The higher in-hospital stroke rate with upfront two-stent PCI warrants similar caution. Dual-stent procedures involved greater anatomical and procedural complexity, more implanted stents, and greater total stent length, while periprocedural MI was also numerically more frequent. EBC MAIN likewise demonstrated greater procedural resource use with systematic dual stenting [5]. However, the small absolute number of strokes and absence of multiplicity adjustment prevent causal attribution; the difference may reflect atherosclerotic burden, procedural complexity, unmeasured risk, or chance. The exclusion of acute coronary syndrome is also relevant when interpreting long-term outcomes. Procedural complexity may also be relevant to periprocedural myocardial injury. In patients with NSTEMI undergoing PCI, bifurcation intervention and total stent length ≥60 mm were independently associated with type 4a MI, which carried adverse prognostic implications [38]. Although our cohort excluded acute coronary syndromes, this evidence provides context for the numerically higher periprocedural MI rate with intended two-stent PCI (6.7% vs. 2.1%; *p* = 0.087), without establishing a causal relationship. Age, LVEF, and SYNTAX score were associated with mortality in the primary model, whereas no statistically significant association was detected for intended strategy. These findings highlight the relevance of patient risk and anatomical complexity but do not establish the relative influence of stenting strategy on survival.

IVUS use was associated with lower mortality in an exploratory adjusted analysis, although the imaging groups differed substantially in anatomy, intended strategy, and procedural characteristics. This association should be interpreted in the context of operator-selected imaging and residual confounding. Earlier LMCA studies support the procedural plausibility of this association. MAIN-COMPARE reported a lower long-term mortality signal with IVUS guidance after propensity matching [39], whereas the NOBLE substudy linked post-PCI IVUS and larger final minimal stent areas to fewer repeat revascularisations [40]. The LMCA substudy of RENOVATE-COMPLEX-PCI demonstrated lower target-vessel failure with intravascular imaging guidance [41], and ROCK II reported fewer target-lesion events after IVUS- or OCT-guided distal LMCA PCI [42]. Conversely, the dedicated randomised OPTIMAL trial did not demonstrate superiority of IVUS guidance for its patient-oriented composite or individual major outcomes [43]. This neutral result does not diminish the value of IVUS for lesion assessment and stent optimisation, but it cautions against attributing an independent survival benefit to imaging itself. IVUS use was operator-selected, and post-stent optimisation was not protocol-mandated. Despite adjustment for selected clinical and anatomical factors, the limited number of deaths and unmeasured treatment-selection factors preclude an inference of causal survival benefit.

The elective IABP comparison included only eight supported and 29 unsupported patients with LVEF ≤40%; bailout cases were excluded. Mortality and MACE were numerically lower with elective support, but no comparison reached statistical significance, and CA-AKI was numerically more frequent. BCIS-1 and LMCA observational studies provide context for selective haemodynamic support [10,11,18,44], whereas IABP-SHOCK II concerned acute MI with cardiogenic shock and is not directly applicable [45]. Given the small, non-randomised groups, these findings remain descriptive and cannot establish benefit or absence of benefit from elective IABP support.

Several features strengthen the clinical relevance of this study, including its well-characterised non-ACS population, 49-month median follow-up, classification by the initially intended strategy, and integration of adjusted survival analysis with anatomy-, technique-, imaging-, and haemodynamic-support–based evaluations. Clinically, the findings favour an individualised approach. Classification according to the initially intended strategy preserved the original procedural decision despite crossover to side-branch stenting, while the combination of multivariable survival analysis with complementary evaluations of true bifurcation anatomy, individual two-stent techniques, IVUS use, ventricular dysfunction, and elective haemodynamic support provided an integrated assessment of both strategic and patient-level determinants of outcome. Stepwise provisional stenting remains an appropriate default when LCx disease is limited, whereas planned two-stent PCI becomes more rational when a large and important LCx contains long or severe ostial disease. DK-crush retains the strongest dedicated randomised evidence for complex distal LMCA bifurcations, although emerging DK-culotte data caution against applying a universal hierarchy across two-stent techniques [6,26,30,31,32,33,34,35,36,37]. IVUS may support anatomical assessment and stent optimisation, while elective haemodynamic support should be reserved for carefully selected patients according to ventricular reserve, jeopardised myocardial territory, and anticipated procedural instability. The principal contribution of this study is therefore not the identification of a universally preferable strategy, but the demonstration that durable LMCA bifurcation PCI depends on aligning patient risk and lesion complexity with an appropriately selected and technically optimised intervention. These findings should nevertheless be interpreted in light of the limitations discussed below.

## 5. Limitations

Several limitations should be acknowledged. First, the retrospective, single-centre design precludes causal inference and may limit the generalisability of the findings. The bifurcation strategy, use of IVUS, and elective haemodynamic support were determined by the treating operators according to clinical and anatomical considerations, introducing selection bias and confounding by indication. In particular, patients selected for upfront two-stent PCI had substantially more complex coronary anatomy.

Despite additional anatomical and propensity-score adjustment, the limited number of deaths constrained model complexity and precision. Side-branch lesion length and reference diameter, bifurcation angle, calcification, detailed procedural optimisation measures, separate IVUS timing categories, and the component-level breakdown of procedural failures were unavailable for analysis. Differences in IVUS use, operator selection, and other unmeasured factors may have contributed to residual confounding. Secondary non-fatal event dates and post-discharge stroke data were also unavailable; crude secondary-event proportions therefore do not account for variable follow-up or competing death. The study-specific MACE definition limits comparison with composites including periprocedural MI or longer-term stroke.

Second, the technique-specific and supportive-treatment analyses were exploratory, largely unadjusted, and limited by small subgroups, particularly for conventional culotte stenting and elective IABP support (8 supported vs. 29 unsupported patients). These findings are therefore imprecise and cannot establish treatment efficacy. IVUS use was also non-protocolised and operator-selected; consequently, its association with improved outcomes remains susceptible to selection bias and unmeasured confounding and should not be interpreted as causal.

Third, the exclusion of patients with acute coronary syndromes, previous CABG, or previous LMCA PCI limits the generalisability of the findings to these populations. Although angiograms were reviewed by two experienced interventional cardiologists, neither independent core-laboratory assessment nor prospective blinded event adjudication was performed, and stent thrombosis was not systematically adjudicated; some misclassification therefore remains possible. Finally, multiple secondary and subgroup comparisons were conducted without adjustment for multiplicity, increasing the likelihood of chance findings. Accordingly, the principal results should be interpreted as associations, while the subgroup findings remain hypothesis-generating and require confirmation in larger prospective multicentre studies.

## 6. Conclusions

In this real-world cohort of patients undergoing PCI for unprotected distal LMCA bifurcation disease outside the setting of acute coronary syndrome, no statistically significant association between initially intended strategy and long-term all-cause mortality was observed. Patients selected for upfront two-stent PCI had substantially greater anatomical complexity. With 46 deaths, the adjusted estimate remained imprecise (HR, 0.83; 95% CI, 0.43–1.60) and does not establish equivalence or exclude clinically meaningful benefit or harm. The higher observed proportions of TLR and in-hospital stroke warrant cautious interpretation in the context of treatment-selection differences and multiple comparisons. The true bifurcation, DK-crush versus conventional culotte, IVUS, and elective-IABP analyses, including the exploratory adjusted IVUS model, remain hypothesis-generating and cannot establish comparative treatment efficacy. The study provides long-term real-world context for individualised distal LMCA PCI, emphasising the collective consideration of lesion complexity, ventricular reserve, procedural technique, imaging guidance, and anticipated haemodynamic needs. Prospective multicentre studies are required to determine whether such tailored procedural planning translates into improved long-term outcomes.

## Figures and Tables

**Figure 1:**
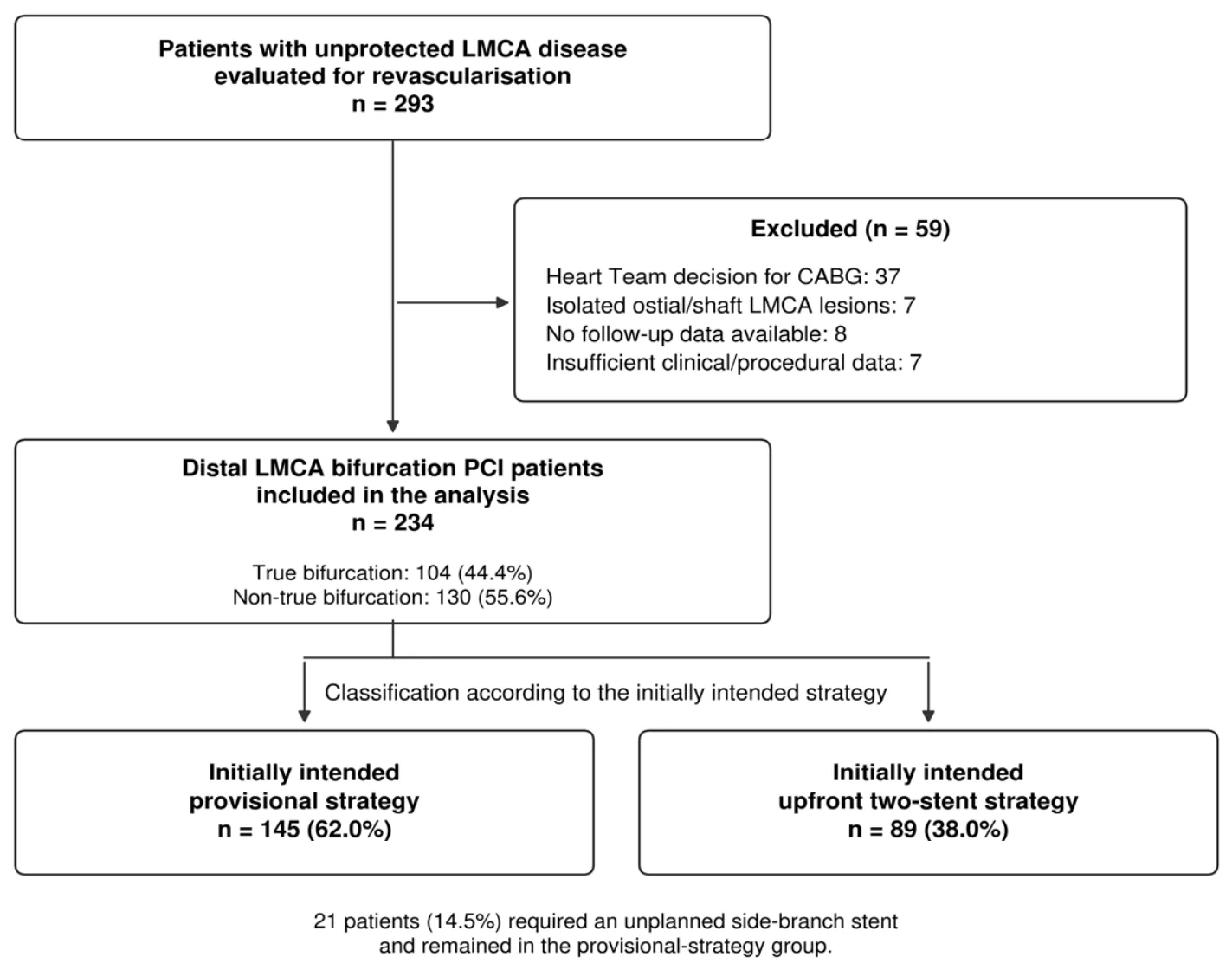
Patient selection and classification according to the initially intended stenting strategy. CABG, coronary artery bypass grafting; LMCA, left main coronary artery; PCI, percutaneous coronary intervention.

**Figure 2:**
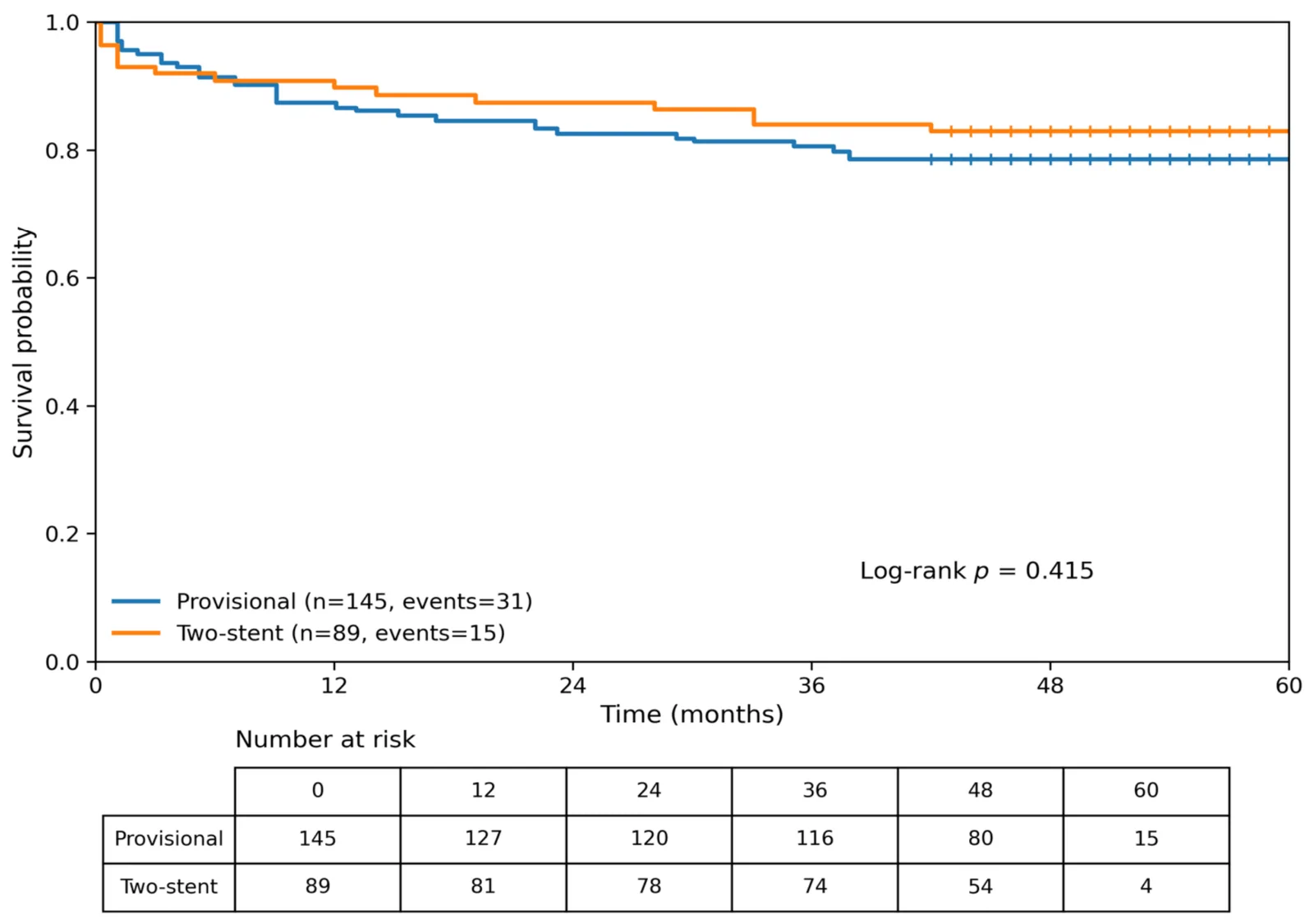
Kaplan–Meier curves for overall survival according to the initially intended bifurcation strategy.

**Figure 3:**
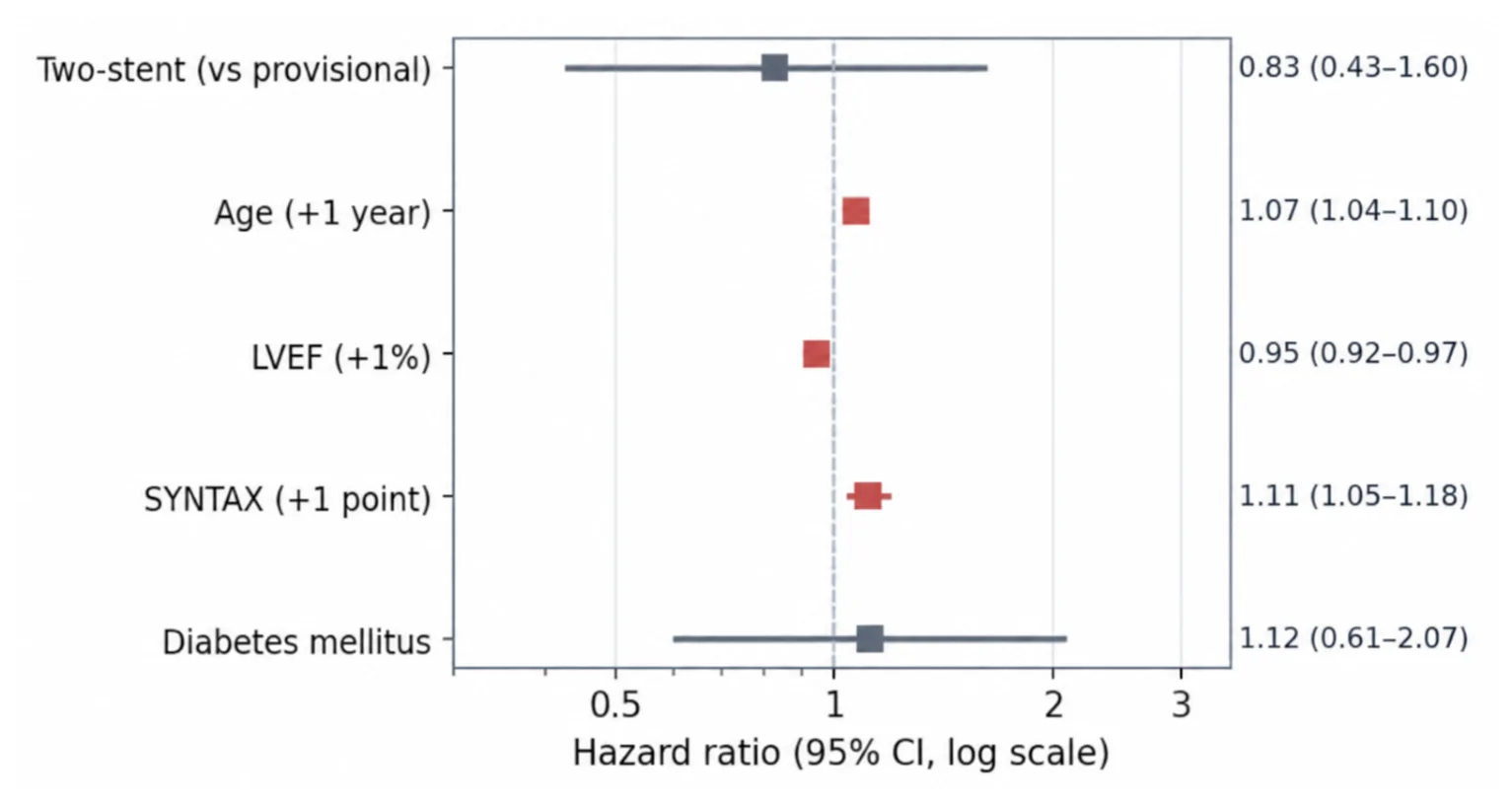
Forest plot of the multivariable Cox proportional-hazards analysis for all-cause mortality.

**Figure 4:**
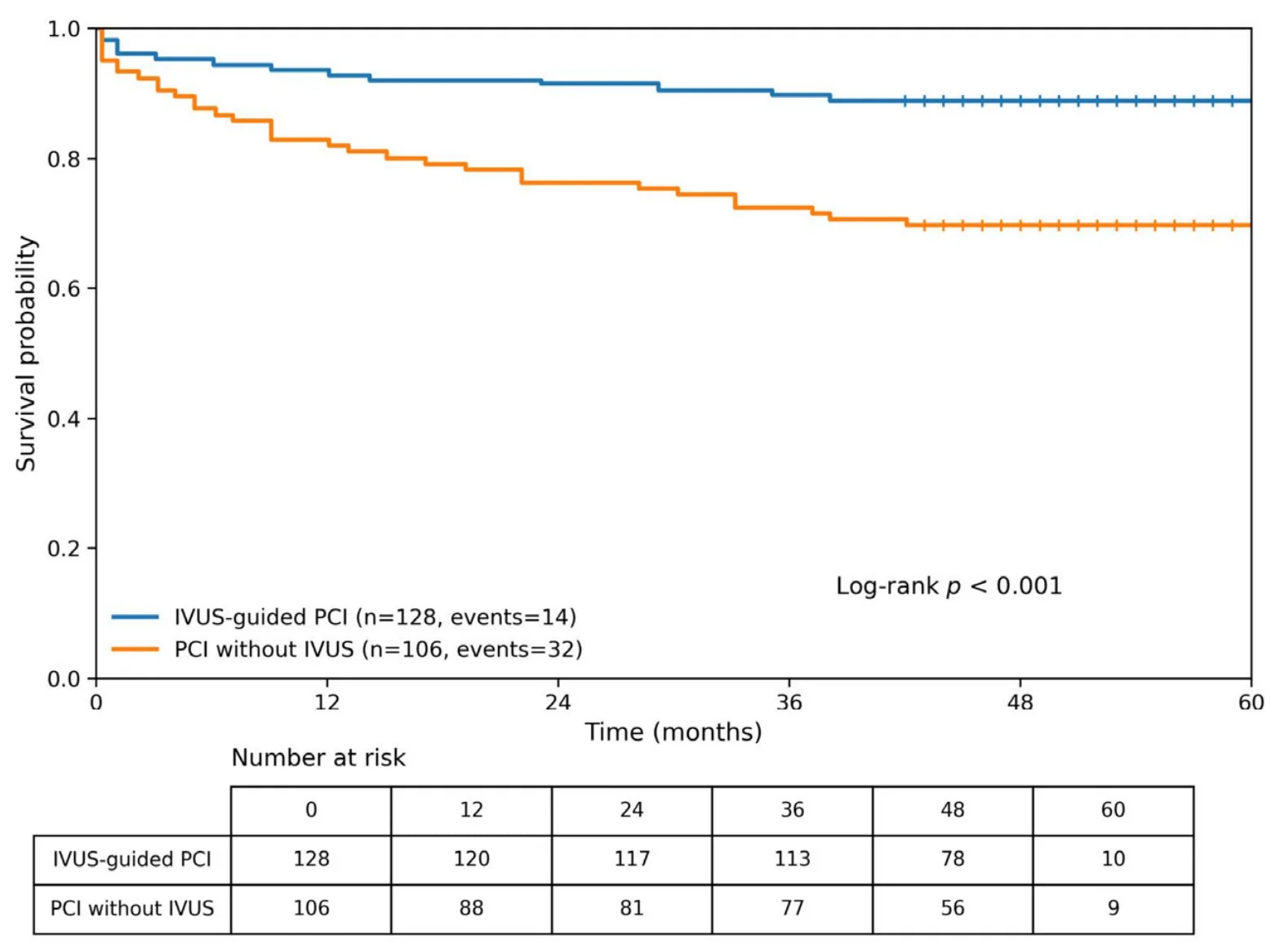
Kaplan–Meier curves for overall survival according to IVUS use. IVUS: intravascular ultrasound.

**Figure 5:**
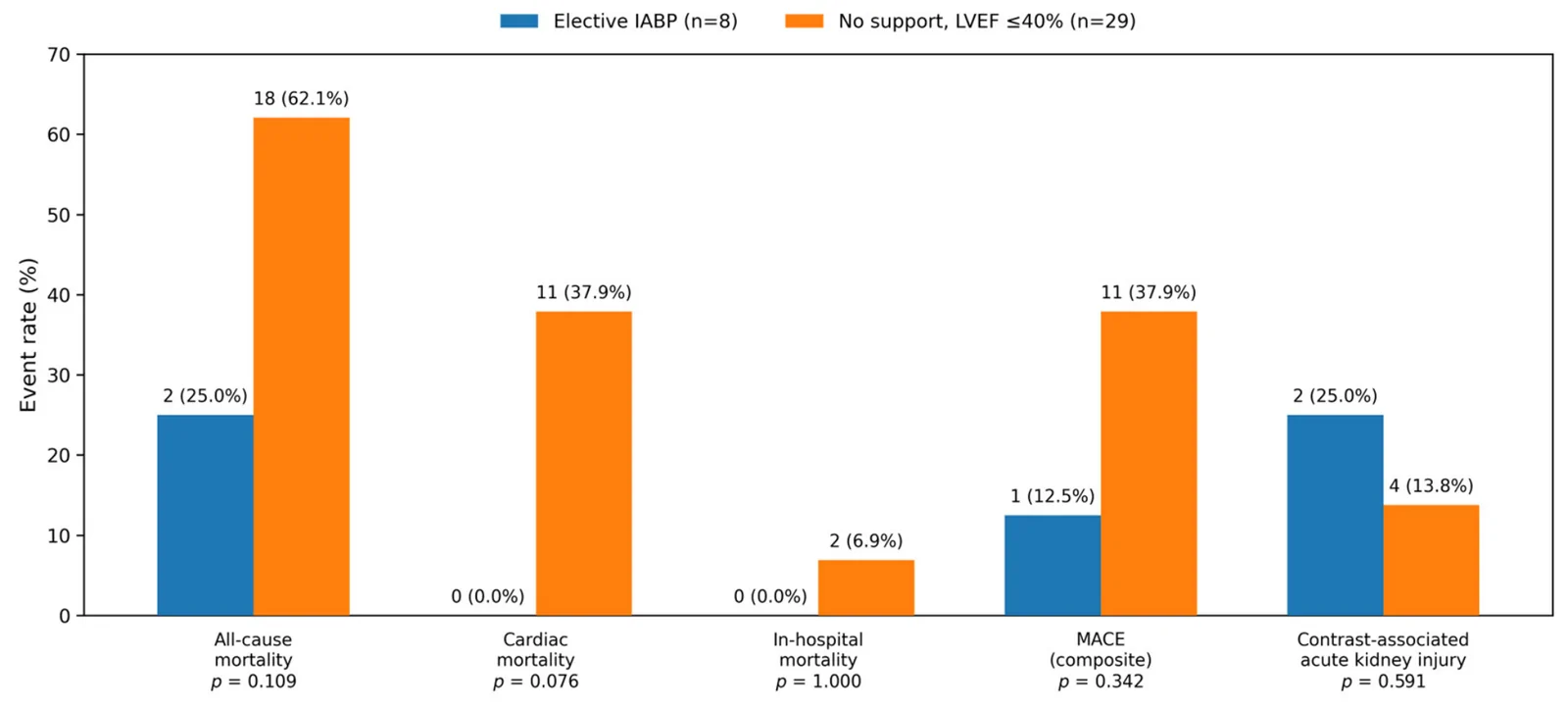
Clinical outcomes according to elective intra-aortic balloon pump support among patients with an LVEF ≤40%. Patients who received bailout IABP support because of intraprocedural haemodynamic deterioration (n = 3) were excluded. Values are presented as n/N (%). All comparisons are exploratory and unadjusted. CA-AKI, contrast-associated acute kidney injury; IABP, intra-aortic balloon pump; LVEF, left ventricular ejection fraction; MACE, major adverse cardiovascular events.

**Table 1:** Baseline and procedural characteristics according to the initially intended bifurcation strategy.

	Provisional Strategy (n = 145)	Two-Stent Strategy (n = 89)	*p* Value
Age, years *	65.6 ± 12.1	64.5 ± 11.4	0.501
Male sex, n (%)	112 (77.2%)	74 (83.1%)	0.358
DM, n (%)	47 (32.4%)	25 (28.1%)	0.582
HT, n (%)	104 (71.7%)	55 (61.8%)	0.151
HL, n (%)	125 (86.2%)	80 (89.9%)	0.532
AF, n (%)	13 (9.0%)	9 (10.1%)	0.951
LVEF, % *	53.3 ± 12.3	56.0 ± 11.1	0.084
LVEF ≤ 40%, n (%)	27 (18.6%)	13 (14.6%)	0.540
eGFR *, mL/min/1.73 m^2^	78.2 ± 25.7	80.2 ± 30.0	0.392
Haemoglobin, g/dL *	12.7 ± 2.0	13.0 ± 1.9	0.375
SYNTAX score *	27.0 ± 6.4	32.1 ± 4.1	**<0.001**
True bifurcation lesion, n (%)	29 (20.0%)	75 (84.3%)	**<0.001**
Radial access, n (%)	121 (83.4%)	68 (76.4%)	0.248
IVUS, n (%)	47 (32.4%)	81 (91.0%)	**<0.001**
Predilatation and lesion preparation, n (%)	145 (100.0%)	89 (100.0%)	1.000
Total stent number *	2.1 ± 0.9	3.1 ± 0.9	**<0.001**
Total stent length, mm *	55.3 ± 26.4	92.1 ± 28.5	**<0.001**
Side-branch stent, n (%)	21 (14.5%)	89 (100.0%)	**<0.001**
Elective IABP, n (%)	6 (4.1%)	2 (2.2%)	0.714
Procedural success, n (%)	132 (91.0%)	82 (92.1%)	0.959
No-reflow, n (%)	5 (3.4%)	0 (0.0%)	0.160

* Data are presented as mean ± standard deviation or n (%); AF: atrial fibrillation, DM: diabetes mellitus, eGFR: estimated glomerular filtration rate, HL: hyperlipidaemia, HT: hypertension, IABP: intra-aortic balloon pump, IVUS: intravascular ultrasound, LVEF: left ventricular ejection fraction, TIMI: Thrombolysis in Myocardial Infarction.

**Table 2:** Clinical outcomes according to the initially intended bifurcation strategy.

Outcome	Provisional Strategy (n = 145)	Two-Stent Strategy (n = 89)	*p* Value
All-cause mortality, n (%)	31 (21.4%)	15 (16.9%)	0.499
Cardiac mortality, n (%)	13 (9.0%)	6 (6.7%)	0.720
In-hospital mortality, n (%)	2 (1.4%)	2 (2.2%)	0.636
Periprocedural MI, n (%)	3 (2.1%)	6 (6.7%)	0.087
Non-fatal MI during follow-up, n (%)	9 (6.2%)	6 (6.7%)	1.000
Target-lesion revascularisation (TLR), n (%)	3 (2.1%)	7 (7.8%)	**0.033**
Target-vessel revascularisation, n (%)	9 (6.2%)	9 (10.1%)	0.316
Contrast-associated acute kidney injury, n (%)	9 (6.2%)	4 (4.5%)	0.771
In-hospital stroke, n (%)	1 (0.7%)	5 (5.6%)	**0.031**
MACE (composite), n (%)	23 (15.9%)	16 (18.0%)	0.810

MACE: major adverse cardiovascular events; MI: myocardial infarction. *p* values were calculated using Pearson’s chi-square or Fisher’s exact test, as appropriate.

**Table 3:** Multivariable Cox proportional-hazards analysis of all-cause mortality.

Variable	Adjusted Hazard Ratio (HR) (95% CI)	*p* Value
Upfront two-stent vs. provisional strategy	0.83 (0.43–1.60)	0.579
Age, per 1-year increase	1.07 (1.04–1.10)	**<0.001**
LVEF, per 1-percentage-point increase	0.95 (0.92–0.97)	**<0.001**
SYNTAX score, per 1-point increase	1.11 (1.05–1.18)	**<0.001**
Diabetes mellitus	1.12 (0.61–2.07)	0.719

CI: confidence interval; HR: hazard ratio; LVEF: left ventricular ejection fraction. The provisional strategy served as the reference category.

**Table 4:** Clinical outcomes according to the initially intended strategy in patients with true bifurcation lesions.

	Provisional Strategy (n = 29)	Two-Stent Strategy (n = 75)	*p* Value
All-cause mortality, n (%)	10 (34.5%)	13 (17.3%)	0.104
Cardiac mortality, n (%)	6 (20.7%)	5 (6.7%)	0.069
TLR, n (%)	0 (0.0%)	7 (9.3%)	0.186
TVR, n (%)	0 (0.0%)	9 (12.0%)	0.059
MACE, n (%)	9 (31.0%)	13 (17.3%)	0.205

Data are presented as n (%). MACE: major adverse cardiovascular events; TLR: target lesion revascularisation; TVR: target vessel revascularisation. This was an exploratory analysis without adjustment for multiple comparisons.

**Table 5:** Baseline characteristics and clinical outcomes according to the two-stent technique.

	DK-Crush (n = 74)	Culotte (n = 15)	*p* Value
Age, years *	65.0 ± 11.8	62.0 ± 9.1	0.411
LVEF, % *	55.3 ± 11.7	59.3 ± 6.2	0.438
SYNTAX score *	32.2 ± 4.2	31.8 ± 4.0	0.757
True bifurcation lesion, n (%)	64 (86.5%)	11 (73.3%)	0.243
Total stent length, mm *	94.4 ± 29.4	80.8 ± 21.4	0.111
All-cause mortality, n (%)	14 (18.9%)	1 (6.7%)	0.248
Cardiac mortality, n (%)	5 (6.8%)	1 (6.7%)	1.000
TLR, n (%)	3 (4.1%)	4 (26.7%)	**0.014**
TVR, n (%)	5 (6.7%)	4 (26.7%)	**0.040**
MACE, n (%)	13 (17.6%)	3 (20.0%)	0.729

* Data are presented as mean ± standard deviation or n (%). This was an exploratory analysis without adjustment for multiple comparisons. MACE: major adverse cardiovascular events, TLR: target lesion revascularisation, TVR: target vessel revascularisation.

**Table 6:** Clinical outcomes according to left ventricular ejection fraction.

	LVEF ≤ 40% (n = 40)	LVEF > 40% (n = 194)	*p* Value
All-cause mortality	22 (55.0%)	24 (12.4%)	**<0.001**
Cardiac mortality	13 (32.5%)	6 (3.1%)	**<0.001**
MACE	15 (37.5%)	24 (12.4%)	**<0.001**

Data are presented as n (%). LVEF: left ventricular ejection fraction; MACE: major adverse cardiovascular events. This was an exploratory analysis without adjustment for multiple comparisons.

**Table 7:** Unadjusted clinical outcomes according to IVUS use.

	IVUS-Guided PCI (n = 128)	PCI Without IVUS (n = 106)	*p* Value
All-cause mortality	14 (10.9%)	32 (30.2%)	**<0.001**
Cardiac mortality	6 (4.7%)	13 (12.3%)	0.061
TLR	6 (4.7%)	4 (3.8%)	0.731
MACE	13 (10.2%)	26 (24.5%)	**0.006**

Data are presented as n (%). IVUS: intravascular ultrasound; MACE: major adverse cardiovascular events; TLR: target lesion revascularisation. This was an exploratory, unadjusted analysis without correction for multiple comparisons.

## Data Availability

The data presented in this study are available on request from the corresponding author due to privacy considerations and institutional restrictions related to potentially identifiable clinical information. The data are not publicly available.

## Supplementary Materials

The following supporting information can be downloaded at: https://www.mdpi.com/article/10.3390/jcm15207817/s1, Table S1: Standardised mean differences in baseline and procedural characteristics according to the initially intended stenting strategy; Table S2: Sensitivity analyses of the association between initially intended stenting strategy and all-cause mortality; Table S3: Characteristics and exploratory adjusted mortality analysis according to IVUS use.
