# Supplementary material for: Long-Term Outcomes According to the Initially Intended Stenting Strategy for Unprotected Distal Left Main Bifurcation PCI: A Single-Centre Retrospective Cohort Study
Source: J Clin Med. 2026 Oct 10;15(20):7817. doi: 10.3390/jcm15207817 (PMC13705433; doi:10.3390/jcm15207817)
Supplement: Supplementary file 1 [file jcm-15-07817-s001.zip › jcm-4576565-supplementary.pdf]

Table S1. Standardised mean differences in baseline and procedural characteristics according to the initially intended stenting strategy

Characteristic	Provisional strategy (n=145)	Upfront two-stent strategy (n=89)	SMD
**Baseline characteristics**			
Age, years	65.6 ± 12.1	64.5 ± 11.4	−0.10
Male sex, n (%)	112 (77.2)	74 (83.1)	0.15
Diabetes mellitus, n (%)	47 (32.4)	25 (28.1)	−0.09
Hypertension, n (%)	104 (71.7)	55 (61.8)	−0.21
Hyperlipidaemia, n (%)	125 (86.2)	80 (89.9)	0.11
Atrial fibrillation, n (%)	13 (9.0)	9 (10.1)	0.04
LVEF, %	53.3 ± 12.3	56.0 ± 11.1	0.23
eGFR, mL/min/1.73 m^2	78.2 ± 25.7	80.2 ± 30.0	0.07
Haemoglobin, g/dL	12.7 ± 2.0	13.0 ± 1.9	0.12
SYNTAX score	27.0 ± 6.4	32.1 ± 4.1	0.95
True bifurcation lesion, n (%)	29 (20.0)	75 (84.3)	1.68
**Procedural characteristics**			
IVUS use, n (%)	47 (32.4)	81 (91.0)	1.51
Total stent number	2.1 ± 0.9	3.1 ± 0.9	1.07
Total stent length, mm	55.3 ± 26.4	92.1 ± 28.5	1.34
Side-branch stent, n (%)	21 (14.5)	89 (100.0)	3.44

Data are presented as mean ± standard deviation or n (%). Positive SMDs indicate higher means or proportions in the upfront two-stent group, whereas negative SMDs indicate higher values in the provisional group. The magnitude of between-group differences was interpreted using the absolute SMD values.

eGFR, estimated glomerular filtration rate; IVUS, intravascular ultrasound; LVEF, left ventricular ejection fraction; SMD, standardised mean difference.

Table S2. Sensitivity analyses of the association between initially intended stenting strategy and all-cause mortality

Analysis	Patients, n	Deaths, n	Adjusted HR for upfront two-stent versus provisional strategy (95% CI)	p value
Primary multivariable Cox model[a]	234	46	0.83 (0.43–1.60)	0.579
Cox model additionally adjusted for true bifurcation status[b]	234	46	0.84 (0.39–1.83)	0.665
Propensity-score-adjusted Cox model, full cohort[c]	234	46	0.71 (0.31–1.62)	0.412
Propensity-score-adjusted Cox model restricted to common support[d]	215	41	0.73 (0.32–1.67)	0.451
Cox model including true bifurcation status and a time-varying age effect[e]	234	46	0.85 (0.39–1.84)	0.679

HR, hazard ratio; CI, confidence interval; LVEF, left ventricular ejection fraction; eGFR, estimated glomerular filtration rate. The provisional strategy was the reference category in all models. Analyses retained the initially intended strategy irrespective of subsequent crossover. Tied event times were handled using the Breslow method.

[a]The primary model included intended stenting strategy, age, LVEF, SYNTAX score, and diabetes mellitus and is reproduced here for comparison.

[b]The primary model was additionally adjusted for true bifurcation status, defined as Medina 1,1,1, 1,0,1, or 0,1,1.

[c]The propensity score for intended upfront two-stent PCI was estimated using logistic regression including age, sex, diabetes mellitus, hypertension, LVEF, eGFR, SYNTAX score, and true bifurcation status. The Cox model included intended strategy and the propensity score as a continuous covariate.

[d]Common support was defined as the intersection of the observed propensity-score ranges in the two strategy groups. The restricted analysis included 131 provisional and 84 upfront two-stent patients. Propensity scores estimated in the full cohort were retained.

[e]The model in row 2 was additionally fitted with an age-by-Kaplan–Meier-transformed-time interaction following a signal of non-proportionality for age.

All p values are two-sided and nominal. These exploratory sensitivity analyses do not establish equivalence or eliminate residual confounding.

Table S3. Characteristics and exploratory adjusted mortality analysis according to IVUS use

Variable	IVUS-guided PCI (n=128)	PCI without IVUS (n=106)	p value
**Baseline and selected procedural characteristics**			
Age, years	65.0 ± 11.7	65.3 ± 12.1	0.915
Male sex, n (%)	100 (78.1%)	86 (81.1%)	0.686
Diabetes mellitus, n (%)	36 (28.1%)	36 (34.0%)	0.412
Hypertension, n (%)	88 (68.8%)	71 (67.0%)	0.882
LVEF, %	55.7 ± 10.9	52.6 ± 12.8	0.046
eGFR, mL/min/1.73 m^2	80.8 ± 27.0	76.8 ± 27.8	0.361
SYNTAX score	29.8 ± 5.6	28.0 ± 6.6	**0.030**
True bifurcation lesion, n (%)	75 (58.6%)	29 (27.4%)	**<0.001**
Initially intended upfront two-stent strategy, n (%)	81 (63.3%)	8 (7.5%)	**<0.001**
Total stent number	2.8 ± 1.0	2.1 ± 0.9	**<0.001**
Total stent length, mm	80.6 ± 31.5	55.6 ± 28.4	**<0.001**
**Exploratory adjusted mortality analysis**			
Adjusted HR for all-cause mortality (95% CI)	0.28 (0.14–0.56)	Reference	**<0.001**

IVUS, intravascular ultrasound; LVEF, left ventricular ejection fraction; eGFR, estimated glomerular filtration rate; HR, hazard ratio; CI, confidence interval. Characteristics are presented as mean ± standard deviation or n (%). Between-group comparisons used the Mann–Whitney U test for continuous variables and the chi-square test with Yates continuity correction for categorical variables.

The exploratory mortality model included all 234 patients and 46 deaths and adjusted for initially intended stenting strategy, age, LVEF, SYNTAX score, diabetes mellitus, and true bifurcation status. An age-by-Kaplan–Meier-transformed-time interaction allowed the age effect to vary with time. Tied event times were handled using the Breslow method. PCI without IVUS was the reference category. Stent number and length were descriptive procedural characteristics and were not included in the adjustment model.

P values are nominal and unadjusted for multiple comparisons. The adjusted association remains susceptible to residual confounding and does not establish a causal survival benefit.
